# Analysis of Biomechanical Properties of the Lumbar Extensor Myofascia in Elderly Patients with Chronic Low Back Pain and That in Healthy People

**DOI:** 10.1155/2020/7649157

**Published:** 2020-02-18

**Authors:** Zugui Wu, Yue Zhu, Wu Xu, Junquan Liang, Yingxin Guan, Xuemeng Xu

**Affiliations:** ^1^The Fifth Clinical College of Guangzhou University of Chinese Medicine, Guangzhou 510000, China; ^2^Baishui Town Health Center, Zhanyi District, Qujing 655000, China; ^3^Guangdong Provincial Second Hospital of Traditional Chinese Medicine, Guangzhou 510000, China; ^4^Guangzhou University of Chinese Medicine, Guangzhou 510000, China

## Abstract

There is limited research on the changes of biomechanical characteristics of the lumbar extensor myofascia in elderly patients with chronic low back pain. This study aimed to compare the biomechanical properties of the lumbar extensor myofascia in elderly patients with chronic low back pain and healthy people when resting and to analyze the relationship between the Japanese Orthopedic Association (JOA) score, visual analog scale (VAS) score, Cobb angle, and disease course and the biomechanical characteristics of the lumbar extensor myofascia. This case-control study included 40 elderly patients with chronic low back pain and 40 healthy volunteers. MyotonPRO was used to measure the biomechanical properties of the bilateral lumbar extensor myofascia (at L3/L4 level) in all participants, and the reliability of the MyotonPRO test was measured. Cobb angle was measured from lumbar computed tomography or magnetic resonance imaging data. JOA and VAS scores were used to evaluate lumbar function and pain. We found that muscle tone, stiffness, and elasticity of the left and right lumbar extensor myofascia in patients with chronic low back pain were very reliable among different operators. The average lumbar extensor muscle tone and stiffness were significantly higher in patients with chronic low back pain than those in healthy controls. The average elasticity of the lumbar extensor myofascia of patients with chronic low back pain was significantly lower than that of the healthy controls. The JOA score was negatively correlated, while the VAS score was positively correlated with the mean values of tone, stiffness, and elasticity of the bilateral lumbar extensor myofascia (logarithmic decrement). Disease course had no significant correlation with muscle tone, stiffness, and elasticity of the lumbar extensor myofascia. No significant correlation was found between Cobb angle and muscle tone, stiffness, and elasticity of the lumbar extensor myofascia in either group.

## 1. Introduction

Chronic low back pain (CLBP) is a global health problem with high incidence, and it is often very detrimental to health, thereby causing substantial economic losses to society [1–3]. Studies have confirmed that lumbar back muscle strength [4] and lumbar lordosis angle [5] are of great importance in maintaining the stability of the lumbar spine. Furthermore, decline in the strength of trunk muscles and increase or decrease in lumbar lordosis angle are related to CLBP [6–8]. Excessive increase or decrease in lumbar lordosis angle will lead to excessive load on the intervertebral joints or intervertebral discs, resulting in worsened low back pain [9, 10]. This suggests that deviation from the normal lumbar lordosis angle plays an important role in CLBP [11].

Although back muscle strength also plays an important role in CLBP [12], the measurement of back muscle strength in patients with CLBP is not entirely objective because of the influence of pain and other factors [6]. In addition to muscle strength, biomechanical properties of muscle are important elements of muscle performance. These properties include muscle tone and stiffness, both of which are thought to be the basis for maintaining muscle contraction and muscle function [13]. The biomechanical properties of muscles and fascia when an individual is resting are the intrinsic characteristics of these tissues, and in the back, these properties play an important role in maintaining the stability of spine [14]. Some studies have reported that the stiffness of the lumbar extensor myofascia in patients with ankylosing spondylitis is greater than that of healthy people of the same age, and the elasticity of the lumbar extensor myofascia in such patients is lower than that in healthy people of the same age [14, 15]. Some studies have also quantitatively analyzed the muscle tone and stiffness of the lumbar and dorsal fascia in adolescent patients with CLBP [16]. Nevertheless, there are only few studies on the biomechanical properties of lumbar muscles and fascia at rest in elderly patients with CLBP [17]. Although CLBP is more commonly diagnosed in younger people, it is still diagnosed mostly in the elderly. There is no previous study we could refer to that analyzed the biomechanical properties of the lumbar myofascial tissue in elderly patients with CLBP, nor did we find any study on the correlation between the lumbar lordosis angle, Japanese Orthopedic Association (JOA) score, and the biomechanical properties of the lumbar extensor myofascia.

The biomechanical properties of the paraspinal muscles and fascia are mostly assessed by manual palpation, and this is used to assist in diagnosis and evaluation of therapeutic effect [18, 19]. However, the reliability of manual palpation in clinical application has always been doubted [20, 21]. Advanced testing techniques such as ultrasound elastography and magnetic resonance elastography are not always feasible because of their high costs and inconvenient operation [22, 23]. Therefore, it has been difficult to quantify the biomechanical properties of muscle tissue clinically.

MyotonPRO is a handheld muscle detector that quickly and noninvasively measures tone, elasticity, and stiffness of the muscles and myofascial tissues [17]. Studies have confirmed that MyotonPRO is accurate and reliable for testing the biomechanical properties of the muscles and myofascial tissues in both healthy and sick people [24, 25]. This includes assessment of the properties of the lumbar and back muscles and myofascial tissues [16, 17].

The Cobb angle is used to assess the degree of lumbar scoliosis in adolescent idiopathic scoliosis and to evaluate the biomechanical properties of the muscle using elastography. The muscle tension and stiffness of the paravertebral vertebrae were higher than those of the convex and concave sides. Hardness was positively correlated with the Cobb angle; however, there was no significant difference in elasticity, and it was concluded that the biomechanical properties of paravertebral muscles were affected by the degree of scoliosis [26]. There are many imaging methods for evaluating the lumbar lordosis angle [27, 28]. The most commonly used method is to measure the Cobb angle of the lumbar spine from lumbar lateral X-ray films taken with the patient standing [28, 29]. Some studies have shown that supine lumbar magnetic resonance imaging (MRI) can replace the upright X-ray imaging for measuring the Cobb angle [30]. In this study, the lumbar Cobb angle of all participants was measured using computed tomography (CT) or lumbar MRI in the supine position.

The purpose of this study was to compare and analyze the tone, stiffness, and elasticity of the lumbar extensor myofascia between elderly patients with CLBP (CLBP group) and healthy people (control group) and to study the relationship between the JOA score, the visual analog scale (VAS) score, and disease course and biomechanical parameters of the lumbar extensor myofascia in patients with CLBP. The differences in lumbar Cobb angles between the CLBP group and the control group were also analyzed, and the correlation between the lumbar Cobb angle and biomechanical parameters of lumbar muscles between the two groups was identified. This study is significant because it provides data regarding the prevention of CLBP as well as a reference for the biomechanical properties of lower-back muscles in elderly patients with CLBP. We hypothesized that the tone and stiffness of the bilateral extensor myofascia would be higher and the elasticity of these tissues would be lower in the CLBP group than in the control group.

## 2. Materials and Methods

### 2.1. Recruitment and Exclusion Criteria

For the test group, 40 elderly patients with CLBP (20 males, 20 females, 50–80 years old) were recruited from the orthopedic clinic of Guangdong Provincial Second Hospital of Traditional Chinese Medicine. For the control group, 40 volunteers (20 males, 20 females, 50–80 years old), who self-reported as healthy, without low-back-related diseases and other diseases that might affect the biomechanical properties of low back muscles, were recruited from neighboring community residents and accompanying family members of patients. A member of the research team was responsible for screening the eligible participants. The recruiting criteria for patients with CLBP were as follows: (1) age 50–80 years; (2) history of low back pain for more than 3 months; and (3) patients who had not received systematic interventional treatment within 4 weeks before being recruited. The exclusion criteria of patients with CLBP were as follows: (1) history of spinal surgery; (2) history of neurological diseases; (3) lumbar diseases other than CLBP, including low back fracture and low back tumors; (4) presence of wounds or scars on the skin of the test site; and (5) body mass index (BMI) ≥30 [16].

### 2.2. Ethical Review

The study was approved by the Ethics Committee of Guangdong Provincial Second Hospital of Traditional Chinese Medicine (No. [2018] 51). The study protocol was strictly consistent with the Helsinki Declaration. We supplied prospective participants with the specific details of the study, and all those who were willing to participate in the study signed a detailed written informed consent document. Moreover, we provided each enrolled participant with an informed consent document, informing them that they had the right to withdraw from the study at any time without any condition.

### 2.3. Process of the MyotonPRO Test

The MyotonPRO test was conducted in the outpatient department of Guangdong Provincial Second Hospital of Traditional Chinese Medicine. The operators received 4 h of professional training from a trainer from Myoton Agent Company, and we also purchased the MyotonPRO equipment from the company. The training included both theoretical and practical sessions. After the training, operators received training for 1 week to learn standardized operation of the equipment.

Before the MyotonPRO test, patients were told to place their hands on both sides of the body to make sure that they were completely relaxed and to avoid physical muscle tone. At the beginning of the test, participants lay prone on the treatment bed, exposing their waist skin. They were asked to hold their breaths for 5 seconds at the end of exhalation to reduce the influence of abdominal pressure on the test. The muscle state was monitored using electromyography (EMG) during the test. The root mean square (RMS) of the myoelectric signal was maintained at 5 *μ*V during the MyotonPRO test, and the muscles were in a resting state before the data being measured using the MyotonPRO; these data were included in the study [17]. Then, the operator made a mark at the point 2 cm from the midpoint of the L3/L4 intervertebral space on both sides of the participants [17, 19] and placed the MyotonPRO probe vertically at the marked points of the lower back (sequence: left side first, then right side). When pressed to the depth required by the device (indicator light changed from red to green), the device sent out mechanical impulses that stimulated the muscle to produce natural damping oscillation. The device calculated the biomechanical parameters of the muscle automatically, including tone, stiffness, and elasticity, after receiving natural damping. During the test, the coefficient of variation (CV) of each test result was observed, and if the CV was more than 3%, the test was repeated. All participants were tested twice, and average results were computed.

### 2.4. Explanation of MyotonPRO Parameters

Damped oscillation frequency (*F*, measured in Hz) represents the muscle tone at rest, and the higher the damped oscillation frequency (Hz), the higher the muscle tone [31–33]. Dynamic stiffness (*S*, measured in N/m) represents muscle stiffness, which reflects the capacity of muscle to resist contraction or external pressure to deform. The higher the dynamic stiffness is, the harder the muscle is [31, 33]. Logarithmic decrement of natural damped oscillation (*D*) is a measure of muscle elasticity. It is expressed only by numerical value, and it has no unit [32, 33]. The elasticity of muscle is inversely proportional to the logarithmic decrement of oscillation amplitude. The lower the logarithmic decrement is, the smaller the dissipation of mechanical energy and the greater the elasticity are.

### 2.5. Measurement of Lower Back Lordosis Angle

**Figure 1:**
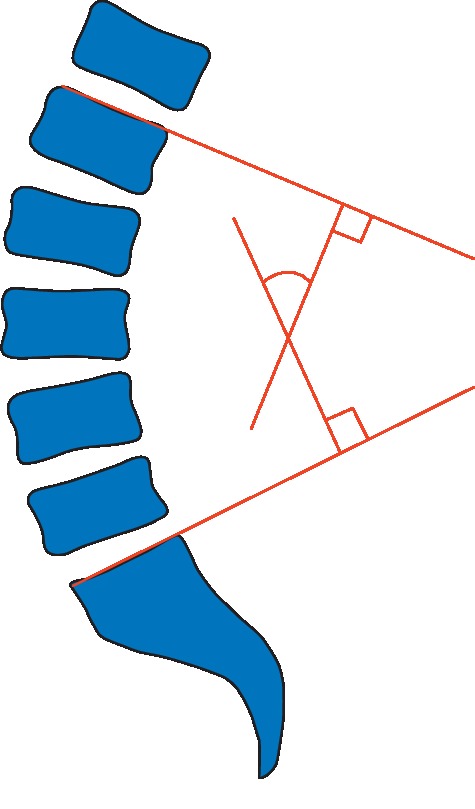
Schematic diagram of the measurement of lumbar lordosis (Cobb) angle.

The angle of lower back lordosis was measured by a physician with 10 years of experience in radiology, using the four-line Cobb method. This method is highly reliable and accurate [34]. A tangent is drawn from the upper border of the L1 vertebra and another from the lower border of the L5 vertebra; then, a vertical line is drawn on each of the two tangents. The angle formed by the intersection of the two vertical lines is the Cobb angle (as shown in Figure 1). CT or MRI data of the lumbar spine were collected in the supine position.

### 2.6. Collection of Basic Data of Participants

This study used the JOA score to assess the degree of lower back vertebra dysfunction, and the VAS score was used to assess the degree of lumbar pain (low back pain was bilateral). The lower spine Cobb angle obtained by CT or MRI was recorded. Simultaneously, the patients' personal information, including age, sex, height, weight, and history of CLBP, was collected.

### 2.7. Statistical Analysis

SPSS version 20.0 (IBM Corp., Armonk, NY, USA) was used for statistical analysis. Shapiro–Wilk test was used to test data normality. Descriptive statistics were carried out for participants' information, such as age, sex, BMI, VAS score, JOA score, disease course, and lower back vertebra lordosis angle. Intraclass correlation coefficient (ICC) was used to evaluate reliability among different operators. ICC values are explained as follows: 0.60 < ICC < 0.75, average; 0.75 < ICC < 0.9, good; and ICC > 0.9, excellent reliability [35]. To evaluate the accuracy of repeated measurements, the standard error of measurement (SEM) and minimum detectable change (MDC) were calculated. Muscle tone, stiffness, and elasticity in the CLBP group and in the control group were compared and analyzed by nonparametric Mann–Whitney *U* test. The correlations of the three parameters of MyotonPRO [muscle tone, stiffness, and elasticity (left side)] with lower back lordosis angle, VAS score, and disease course were analyzed by Spearman correlation test. Pearson's analysis was used to calculate the correlation between the JOA score of patients with CLBP, the Cobb angle of the lumbar spine in healthy people, and the muscle tone, stiffness, and elasticity (right side) of the lumbar extensor myofascia. The level of significance level (P) was set to 0.05.

## 3. Results

### 3.1. Demographic Characteristics

No statistical difference was found between the demographic characteristics of the CLBP group and control group. Moreover, no statistical difference was noted between men and women with CLBP (Table 1).

### 3.2. Reliability Test

The muscle tone, stiffness, and elasticity of the left and right extensor myofascial tissues in the CLBP group were very reliable among different operators. The following values were obtained for muscle tone on the left side, ICC = 0.94, SEM = 0.53, and MDC = 1.47; for stiffness on the left side, ICC = 0.94, SEM = 18.12, and MDC = 50.23; for elasticity on the left side, ICC = 0.90, SEM = 0.09, and MDC = 0.25; for muscle tone on the right side, ICC = 0.92, SEM = 0.59, and MDC = 1.64; for s tiffness on the right side, ICC = 0.95, SEM = 17.03, and MDC = 47.20; and for elasticity on the right side, ICC = 0.94, SEM = 0.07, and MDC = 0.19 (Table 2).

### 3.3. Analysis of the Biomechanical Properties of the Lumbar Extensor Myofascia

The muscle tone of the lumbar extensor myofascia in the CLBP group and control group was assessed. The results showed that the muscle tone of the lumbar extensor myofascia on the left and right sides of the CLBP group was significantly higher than that of the control group (left side, *P*=0.003; right side, *P*=0.010). The average muscle tone on the left side was 16.45 ± 2.14 Hz in the CLBP group vs. 15.15 ± 1.46 Hz in the control group. The average muscle tone on the right side was 16.33 ± 2.07 Hz in the CLBP group vs. 15.21 ± 1.68 Hz in the control group. When the muscle tone measurements of the left and right sides of the lumbar extensor myofascia were averaged; the average muscle tone on both sides was 16.39 ± 2.07 Hz in the CLBP group vs. 15.18 ± 1.49 Hz in the control group. The average muscle tone of the CLBP group was significantly higher than that of the control group (*P*=0.006). There was no significant difference in muscle tone between the left and right sides of the CLBP and control groups (*P* > 0.05).

The stiffness of the lumbar extensor myofascia in the CLBP group and control group was also tested. The results showed that the stiffness of the lumbar extensor myofascia on the left and right sides of CLBP group was significantly higher than that of the control group (left side, *P*=0.002; right side, *P*=0.018). The average stiffness of the left side was 319.66 ± 73.47 N/m in the CLBP group vs. 273.53 ± 44.53 N/m in control group, while the average stiffness of the right side was 318.77 ± 75.67 N/m in the CLBP group vs. 280.57 ± 49.35 N/m in the control group. The average value of bilateral muscle stiffness in the CLBP group was significantly higher than that of the control group (*P*=0.006): the average value of bilateral stiffness was 319.21 ± 73.75 N/m in the CLBP group vs. 277.05 ± 44.70 N/m in the control group. No significant difference was found between the left and right myofascia stiffness in the CLBP group and that in the control group (*P* > 0.05).

The elasticity of the lumbar extensor myofascia in the CLBP group and control group was also tested. The result showed that the elasticity of the lumbar extensor myofascia on the left and right sides of the CLBP group was significantly lower than that of the control group (left side, *P*=0.001; right side, *P*= 0.013), and the average logarithmic decrement of the left side was 1.48 ± 0.30 in the CLBP group vs. 1.27 ± 0.19 in the control group and on the right side was 1.45 ± 0.30 in the CLBP group vs. 1.30 ± 0.17 in the control group. After the logarithmic decrements measured on the left and right sides were averaged, we found that the average value of logarithmic decrement of the lumbar extensor myofascia on both sides of the CLBP group was significantly higher than that of the control group (*P*= 0.002), and the average value of the logarithmic decrement on both sides was 1.47 ± 0.28 in the CLBP group vs. 1.28 ± 0.16 in the control group. No significant difference in the logarithmic decrement of right and left sides was noted between the CLBP group and the control group (*P* > 0.05).

The results of the analyses of biomechanical properties are presented in Table 3.

### 3.4. Correlation Analysis of JOA Score, VAS Score, Disease Course, Cobb Angle, and Biomechanical Parameters of the Lumbar Extensor Myofascia

Table 4 shows the statistical characteristics of the JOA score, VAS score, disease course, and Cobb angle. At the same time, sex differences were calculated for men and women. No significant difference was found between the VAS score, VAS score, disease course, and Cobb angle (*P* > 0.05).  Table 5 shows the correlation between JOA score, VAS score, Cobb angle, disease course, and muscle tone, stiffness, and elasticity of the lumbar extensor myofascia. In the 40 patients with CLBP, the JOA score was significantly related to the mean values of the muscle tone, stiffness, and elasticity of the bilateral lumbar extensor myofascia (muscle tone: *r* = −0.621, *P*=0.01; stiffness: *r* = −0.682, *P*=0.01; and elasticity: *r* = −0.359, *P*=0.023). The VAS score was also significantly correlated with the mean tone, stiffness, and elasticity (muscle tone: *r* = 0.695, *P*=0.01; stiffness: *r* = 0.715, *P*=0.01; and elasticity: *r* = 0.525, *P*=0.001). Disease course and Cobb angle and the average muscle tone, stiffness, and elasticity of the lumbar extensor myofascia on both sides were not significantly correlated (*P* > 0.05). In the control group of 40 healthy people, no significant correlation was noted between the Cobb angle and the mean values of muscle tone, stiffness, and elasticity of the lumbar extensor myofascia (*P* > 0.05). Moreover, no significant difference was found in the lumbar Cobb angle between the CLBP group and the control group (*P* > 0.05) (Tables 4 and 5).

## 4. Discussion

### 4.1. Reliability Test

In this study, we performed a reliability analysis of the MyotonPRO test in patients with CLBP. The results showed that the MyotonPRO equipment had high reliability in testing the biomechanical properties of the lumbar extensor myofascia among different operators (ICC > 0.90). The result is consistent with the result obtained in the reliability test using previous MyotonPRO equipment for testing healthy elderly people and people with diseases, showing good-to-excellent reliability for both patients and healthy people [24, 36]. The reliability of the paraspinal muscle tone and stiffness in adolescents with CLBP and healthy people was also studied. The results also showed that the MyotonPRO device has good reliability in testing the biomechanical properties of the lumbar muscle [16, 37]. Reliability is very important when using instruments in clinical evaluation or research environments. Based on the high reliability results of the MyotonPRO equipment, we can conclude that the evaluation of muscle tone, stiffness, and elasticity using the MyotonPRO equipment is objective and reliable.

### 4.2. Influence of CLBP on Biomechanical Properties of the Lumbar Extensor Myofascia

In this preliminary study, compared with healthy subjects of the same age, the quantified muscle tone and stiffness of lumbar extensors myofascia in elderly patients with CLBP were higher, and elasticity was lower (the logarithmic decrement was higher). This finding confirms our hypothesis and is consistent with the conclusion of other studies that patients with other skeletal muscle-related diseases have higher muscle tone and stiffness and lower elasticity [15, 38, 39]. However, the mechanism of high muscle tone and stiffness and low elasticity of the lower back myofascia in patients with CLBP is still unclear. These parameters may result from the dysfunction of the lower back muscle and fascia. Muscle tone refers to the degree of muscle tone when skeletal muscle is relaxed. The most significant and direct factor affecting muscle tone is muscle contraction [40]. No study has reported that the muscle tone and stiffness in patients with CLBP are higher than those in healthy people of the same age; nevertheless, it can be seen from the research results that the muscle tone and stiffness of patients with CLBP were higher than those of healthy people of the same age and even higher than those of healthy people [17, 37]. Kim et al. found that higher muscle tone indicated greater severity of pain or increased exercise load [41]. Previous studies have found that dysfunctional muscles and fascia have potential pain-producing mechanisms and may be the main source of low back pain [42–44]. Other studies have found that the muscle tone and stiffness of lower back muscles and fascia in patients with CLBP are significantly different from those of healthy people [17, 45], which may be attributed to the underlying pathology and symptoms [46].

Elasticity is an indicator of the ability of muscles to return to their original state after being squeezed or contracted [47]. In the present study, the elasticity of the lumbar extensor myofascia on both sides in patients with CLBP was lower than that in healthy controls (*P* < 0.01). Previous studies on the biomechanical characteristics of lumbar muscles have focused on muscle tone and stiffness, and relatively few studies on elasticity. White et al. used MyotonPRO to analyze the biomechanical characteristics of lumbar muscles (L3-4 levels) in 24 patients with ankylosing spondylitis (average age of 30 years) and 24 age-sex-matched healthy controls. The logarithmic attenuation value of the lumbar muscles (L3-4 level) of the CLBP group was higher than that of the control group, and the elasticity was worse than that of the control group, which is consistent with the result of the present study [14].

Stiffness refers to the ability of muscles to resist contraction or external pressure and keep their original shape unchanged [33]. This study found that the stiffness of the lumbar extensor myofascia on both sides in the CLBP group was higher than that of the control group (*P* < 0.01). Muscle stiffness is caused by a lack of contractile activity [48]. A previous study found that the transverse bridge of muscle fibers changes with long-term contraction of muscles and lacks active or passive motion. With the disappearance of sarcomeres, the stiffness value of muscles will become higher [49]. By analyzing passive elbow movement in Parkinson's disease patients, Watts found that passive muscle stiffness is related to muscle atrophy [50]. Other studies have shown that the process of muscle atrophy has some influence on skeletal muscle stiffness [51, 52]. Previous studies have shown that CLBP causes increased stiffness in the muscle fibers of the waist, which is consistent with the conclusions of the present study [53]. Park et al. [48] also analyzed the biomechanical properties of the suboccipital and superior trapezius muscles in 20 patients with cervicogenic headache and 20 healthy people. They found that the average muscle tone of the suboccipital muscle was 15.6 Hz, the average stiffness was 323.9 Nm, and the average elasticity value was 1.3. The average muscle tone of the upper trapezius muscle was 19.4 Hz, and the average stiffness was 355.5 Nm. The average elasticity value was 1.1. The results were less different from those of the present study; data of healthy people in the control group were also less different from those of the present study. They found that muscle tone and stiffness of the suboccipital and superior trapezius muscles in patients with cervicogenic headache were significantly higher than those in the control group, which is also consistent with the results of the present study [48].

### 4.3. Relationship between the JOA Score, VAS Score, Cobb Angle, and Disease Course, and Biomechanical Properties of the Lumbar Extensor Myofascia

In this study, the correlation between the JOA score, VAS score, Cobb angle, and disease course and muscle tone, stiffness, and elasticity of the lumbar extensor myofascia was analyzed. In the CLBP group, we found that the JOA score negatively correlated with the muscle tone and stiffness of the lumbar extensor myofascia and positively correlated with muscle elasticity (negatively correlated with the logarithmic decrement). The lower the JOA score, the higher the muscle tone and stiffness of lumbar extensor myofascia and the lower the elasticity. The VAS score positively correlated with muscle tone and stiffness of the lumbar extensor myofascia and negatively correlated with muscle elasticity (positively correlated with logarithmic decrement). The higher the VAS score, the higher the muscle tone and stiffness of the lumbar extensor myofascia and the lower the elasticity. The Cobb angle and disease course had no significant correlation with muscle tone, stiffness, and elasticity of the lumbar extensor myofascia (*P* > 0.05). In the control group, we also found no significant correlation between the Cobb angle and the muscle tone, stiffness, and elasticity of the lumbar extensor myofascia (*P* > 0.05). In addition, we found no significant correlation between the Cobb angle and muscle tone, stiffness, and elasticity. This may be due to the small sample size of this study (in the included samples, the Cobb angle range was too small), or there may be other factors that comprehensively affect the biomechanical properties of the lumbar extensor myofascia, such as pain, disease duration, and exercise, rather than the Cobb angle alone. It can thus be concluded that the comprehensive function of the lower back spine and the pain that results from dysfunction are correlated with the biomechanical properties of the lumbar extensor myofascial; however, the specific underlying mechanism needs to be further studied.

At the same time, we found that the Cobb angle of the CLBP group was not different from that of the control group. Murrie et al. studied the lumbar lordosis of the CLBP group and control group and concluded that there was no significant difference between the lumbar lordosis in the CLBP group and control group. Their conclusion is consistent with that of the present study [54]. However, the specific reason for the difference is not clear. Shortz and Haas investigated 352 patients with CLBP and found no correlation between the Cobb angle and pain level in patients with CLBP [55]. Hansen et al. found that changes in lumbar lordosis may not be related to pain and may be related to the natural deterioration of the disc with age [56].

### 4.4. Limitations of the Research

This study has some limitations. First, generally, the depth that the MyotonPRO equipment can reach is not more than 2 cm [15], and lower back muscles, which are located more than 2 cm deep, are more important than the superficial muscles in maintaining the stability of the lower spine [57, 58]. Deep muscles can be studied with magnetic resonance elastography or ultrasonic shear wave elastography. We are therefore planning to carry out relevant research with ultrasound elastography technology. Second, during the MyotonPRO test, although we used EMG to monitor the state of the muscle, RMS data of the EMG signal were not included in the results of this study and the EMG signal was not analyzed for its biomechanical properties. Therefore, we plan to conduct a related study using EMG technology. Third, because the biomechanical characteristics of the lumbar muscles were measured in the prone position, it was not possible to assess the stability of spine during the test period. Fourth, there was a wide range of patients' ages, i.e., 50–80 years. This affects the homogeneous nature that should characterize this study. Fifth, we were unable to strictly control the variables affecting the tone, stiffness, and elasticity of the lumbar extensor myofascia, such as the amount of exercise before the test and the lower back lordosis angle during the test. These factors may affect the parameters of the lumbar extensor myofascia. Finally, this study only tested the tone, stiffness, and elasticity of the lumbar extensor myofascia at the L3/L4 intervertebral space level; it did not test those parameters at other locations on the lumbar spine.

### 4.5. Research Directions in the Future

To determine the changes in lumbar muscle performance in the development of CLBP and carry out quantitative study simultaneously, these researchers will conduct multilevel research on lumbar muscles from multiple perspectives, with surface electromyography and ultrasound elastography, including deep muscles in the lower back. The future study should include a large number of patients with CLBP and should involve patients with CLBP of different age groups and different disease stages. Simultaneously, we can test other locations on the back of patients with CLBP to evaluate the performance of lower back muscles more comprehensively. MyotonPRO equipment is becoming increasingly used clinically, and an increasing number of researchers use it in the detection of muscle-related diseases, such as Parkinson's disease and ankylosing spondylitis [14, 38]. It is very necessary to detect its application in different skeletal muscle-related diseases.

## 5. Conclusions

This study compared the biomechanical properties of the lumbar extensor myofascia in patients with CLBP and in healthy patients. We found that the tone and stiffness of the lumbar extensor myofascia on both sides in patients with CLBP were higher than those in healthy controls, while the elasticity was lower than that in healthy controls. We also found that the JOA score was negatively correlated with the tone and stiffness of the extensor myofascia bilaterally in patients with CLBP and positively correlated with elasticity. The VAS score was positively correlated with the tone and stiffness of the lumbar extensor myofascia on both sides and negatively correlated with elasticity in patients with CLBP. Moreover, no significant correlation was found between the Cobb angle and disease course and muscle tone, stiffness, and elasticity of the lumbar extensor myofascia. As this study only involved 40 patients and 40 healthy controls, research with larger sample sizes is needed in the future to form a complete database and further study the correlation between these factors and other influencing factors.

## Figures and Tables

**Table 1:** Demographic characteristics of the study sample.

Characteristic	CLBP group		Control group		Consolidated gender	
Sex	Male	Female	Male	Female	CLBP	Control group
Age (years)	61.45 ± 8.56	65.30 ± 8.02	63.45 ± 6.17	63.80 ± 7.68	63.37 ± 8.42	63.62 ± 6.88
Height (cm)	169.05 ± 6.63	155.30 ± 6.62	166.90 ± 6.69	157.95 ± 5.76	162.17 ± 9.55	162.42 ± 7.65
Weight (kg)	67.73 ± 7.55	58.47 ± 7.18	66.52 ± 9.82	58.97 ± 6.53	63.10 ± 8.65	62.74 ± 9.08
BMI (kg/m^2^)	23.80 ± 2.62	24.24 ± 2.52	23.79 ± 2.68	23.56 ± 2.66	24.02 ± 2.55	23.67 ± 2.64

*Note*. Each value is presented as mean ± standard deviation. CLBP: chronic low back pain.

**Table 2:** Reliability test.

Side	Parameter	ICC (95% CI)	SEM	MDC
Left	Muscle tone	0.94 (0.89–0.96)	0.53	1.47
	Stiffness	0.94 (0.90–0.97)	18.12	50.23
	Logarithmic decrement	0.90 (0.83–0.95)	0.09	0.25

Right	Muscle tone	0.92 (0.85–0.95)	0.59	1.64
	Stiffness	0.95 (0.90–0.97)	17.03	47.20
	Logarithmic decrement	0.94 (0.89–0.96)	0.07	0.19

*Note*. SEM = SD ∗ √(1–ICC); MDC_95_ = 1.96 ∗ √2 ∗ SEM. ICC: intraclass correlation coefficient; MDC: minimum detectable change; and SEM: standard error of measurement.

**Table 3:** Statistical results of biomechanical properties (muscle tone, stiffness, and elasticity) of the lumbar extensor myofascia in the CLBP group and control group.

			CLBP	Control participant	*P* value
Left	Muscle tone	Mean ± SD	16.45 ± 2.14	15.15 ± 1.46	0.003
		Range	(11.75–20.20)	(12.55–19.30)	
	Stiffness	Mean ± SD	319.66 ± 73.47	273.53 ± 44.53	0.002
		Range	(172.00–470.50)	(182.50–375.50)	
	Logarithmic decrement	Mean ± SD	1.48 ± 0.30	1.27 ± 0.19	0.001
		Range	(1.07–2.34)	(0.96–1.70)	

Right	Muscle tone	Mean ± SD	16.33 ± 2.07	15.21 ± 1.68	0.010
		Range	(12.50–20.20)	(11.00–19.45)	
	Stiffness	Mean ± SD	318.77 ± 75.67	280.57 ± 49.35	0.018
		Range	(184.00–480.50)	(180.00–417.50)	
	Logarithmic decrement	Mean ± SD	1.45 ± 0.30	1.30 ± 0.17	0.013
		Range	(0.95–2.19)	(0.91–1.70)	

Bilateral average	Muscle tone	Mean ± SD	16.39 ± 2.07	15.18 ± 1.49	0.006
		Range	(12.13–20.10)	(11.78–18.75)	
	Stiffness	Mean ± SD	319.21 ± 73.75	277.05 ± 44.70	0.006
		Range	(178.00–475.50)	(194.00–382.00)	
	Logarithmic decrement	Mean ± SD	1.47 ± 0.28	1.28 ± 0.16	0.002
		Range	(1.02–2.27)	(0.95 ± 1.62)	

SD: standard deviation; CLBP: chronic low back pain. *P* values were derived from nonparametric Mann–Whitney *U* test results for the CLBP group and control group.

**Table 4:** Disease course, JOA score, VAS score, and Cobb angle between the CLBP group and control group.

	Factor	Male	Female	Consolidated sex	*P* value
CLBP group	Disease course	34.55 ± 43.10	65.15 ± 53.12	49.85 ± 50.20	0.057
	JOA score	18.10 ± 3.59	19.80 ± 3.33	18.95 ± 3.52	0.080
	VAS score	4.50 ± 1.35	4.00 ± 1.41	4.25 ± 1.39	0.218
	Cobb angle	40.80 ± 7.92	40.25 ± 10.83	40.52 ± 9.37	0.735

Control group	Cobb angle	39.60 ± 9.48	39.00 ± 7.84	39.30 ± 8.59^&^	0.432

*Note*. Values are presented as mean ± standard deviation. CLBP: chronic low back pain; JOA: Japanese Orthopedic Association; and VAS: visual analog scale. *P* value was derived from nonparametric Mann–Whitney *U* test for men and women of the CLBP group. *Note*. ^&^indicates no statistically significant difference.

**Table 5:** Correlation between the JOA score, VAS score, Cobb angle, and disease course, and biomechanical properties of the lumbar extensor myofascia.

CLBP group	Factor	Muscle tone		Stiffness		Logarithmic decrement	
		*r*	*P*	*r*	*P*	*r*	*P*
	JOA score	−0.621	0.01^#^	−0.682	0.01^#^	−0.359	0.023^#^
	VAS score	0.695	0.01^#^	0.715	0.01^#^	0.525	0.001^#^
	Cobb angle	−0.232	0.149	−0.261	0.104	−0.146	0.369
	Duration of disease	0.029	0.861	0.013	0.938	0.115	0.479

Control group	Cobb angle	−0.032	0.843	−0.022	0.895	0.075	0.646

*Note*. ^#^indicates statistically significant difference. CLBP: chronic low back pain; JOA: Japanese Orthopedic Association; and VAS: visual analog scale.

## Data Availability

The research data used to support the findings of this study are included within the Supplementary Materials.

## Supplementary Materials

Supplementary Materials Supplementary materials include a data set for this study, including participants in the chronic low back pain group and the control group. The data collection method has been described in detail in the manuscript, and the relevant results obtained have been discussed in the manuscript. According to the requirements of the ethics committee, the author has anonymized the data set to protect participants' privacy. There are no other restrictions on access to the data set.
